# Signal transduction in cells of the immune system in microgravity

**DOI:** 10.1186/1478-811X-6-9

**Published:** 2008-10-28

**Authors:** Oliver Ullrich, Kathrin Huber, Kerstin Lang

**Affiliations:** 1 Institute of Anatomy, Faculty of Medicine, University of Zurich, Switzerland; 2 Institute of Mechanical Engineering, Faculty of Mechanical Engineering, Otto-von-Guericke University Magdeburg, Germany; 3 Institute of Immunology, Faculty of Biosciences, Witten/Herdecke University, Germany

## Abstract

Life on Earth developed in the presence and under the constant influence of gravity. Gravity has been present during the entire evolution, from the first organic molecule to mammals and humans. Modern research revealed clearly that gravity is important, probably indispensable for the function of living systems, from unicellular organisms to men. Thus, gravity research is no more or less a fundamental question about the conditions of life on Earth. Since the first space missions and supported thereafter by a multitude of space and ground-based experiments, it is well known that immune cell function is severely suppressed in microgravity, which renders the cells of the immune system an ideal model organism to investigate the influence of gravity on the cellular and molecular level. Here we review the current knowledge about the question, if and how cellular signal transduction depends on the existence of gravity, with special focus on cells of the immune system. Since immune cell function is fundamental to keep the organism under imnological surveillance during the defence against pathogens, to investigate the effects and possible molecular mechanisms of altered gravity is indispensable for long-term space flights to Earth Moon or Mars. Thus, understanding the impact of gravity on cellular functions on Earth will provide not only important informations about the development of life on Earth, but also for therapeutic and preventive strategies to cope successfully with medical problems during space exploration.

## The "immune problem" in space

Early reports about disturbed immune cell function in space date back in the 70ties, where reduced reactivity of blood lymphoid cells has been discovered in crew members of Soyuz spaceships and of Skylab and Apollo [1,2]. Recently, a subclinical re-activation varicella zoster virus (VZV) has been reported in astronauts [3,4], a virus which becomes latent in the nervous system after primary infection, but is reactivated frequently in immune suppressed individuals, such as after organ transplantation, and in patients with cancer or AIDS. Whereas it is well known that gravity can be perceived by gravireceptors (statocyst-like organelles or gravisensitive ion channels in the cell membrane) in unicellular organisms such as Paramecium and Loxodes, where it strongly influences intracellular signal transduction and behaviour [5,6], the molecular mechanisms of gravisensitivity in mammalian cells are widely unknown. After the pioneering discovery of Cogoli et al. at the first Spacelab-Mission 20 years ago [7], it is known that proliferative response of lymphocytes after mitogenic stimulation is suppressed in microgravity [8]. In follow-up experiments in order to verify the result from Spacelab 1, it has been demonstrated clearly that factors other than microgravity can be excluded to be responsible for the depressed activation of lymphocytes. Whereas the phenomenon of reduced activation of T cells during microgravity is well described [9,10] and verified, the exact molecular mechanisms are not elucidated.

## Signal transduction and cell-cell communication is disturbed in microgravity

Several investigations evidence alterations in signal transduction in lymphocytes. In lymphocytes, microgravity affected the protein kinase C [11,12] whereas delivery of first activation signal, patching and capping of conA-binding membrane proteins occurred normally in spaceflight [13]. These findings suggest the existence of gravisensitive cellular targets upstream from PKC and downstream from the TCR/CD3, where the lipid-raft-associated membrane-proximal signalosome complex is located. DNA array analysis of T cells subjected to simulated microgravity provided by the random-positioning machine (RPM) revealed an alteration of several signal moduls, in particular NF-kB and MAPK-signaling [14]. Also the expression of the early oncogenes *c-fos, c-myc *and *c-jun *is inhibited during spaceflight [summarized in [15]].

In other studies, gravisensitivity of *pro- and antiapoptotic pathways *has been reported in human mononuclear cells [16], human ML-1 thyroid-carcinoma cells [17] and astrocytes [18] in simulated microgravity. On the molecular level, simulated microgravity induced fas, p53 and bax and reduced bcl-2 [17,19]. Interestingly, the expression of fas was elevated in Jurkat-T-cells also during space flights of the shuttle missions STS-80 and STS-95 [20], suggesting an enhanced fas-fasL-mediated apoptosis of immune cells. During a 14-days space flight (SLS-2-mission) an accumulation of p53 has been found in keratinocytes and myocytes, indicating that central regulatory molecules of nuclear signal transduction and cell cycle are influenced by gravity [21]. The diminished proliferative response of T cells upon stimulation during microgravity could also be caused by a reduced expression of IL-2 receptor as demonstrated in simulated microgravity [22,23], resulting in an impairment of positive regulatory feedback loops. Overall, a decreased capacity of T-cells for the production of cytokines is a prominent effect of microgravity on leukocytes during spaceflight [24].

**Figure 1:**
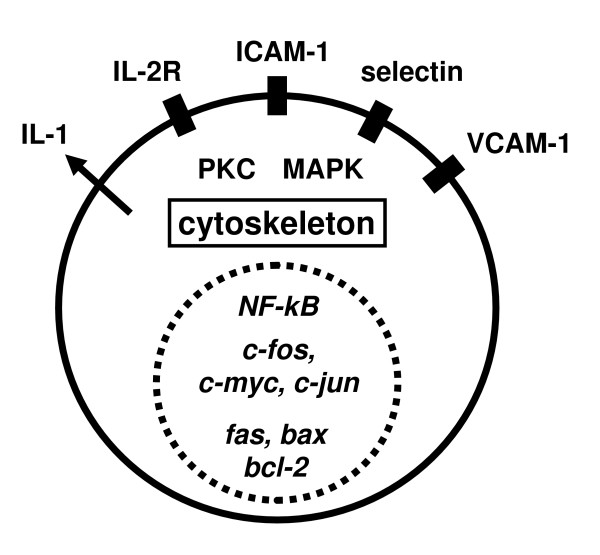
**Gravi-sensitive signal transduction elements in mammalian cells**. Gravi-sensitive signal transduction elements has been detected at the cell surface, such as VCAM-1 (Vascular cell adhesion molecule 1), ICAM-1 (Intercellular adhesion and molecule 1) and IL-2R (interleukin-2 receptor), in the cytoplasma such as PKC (protein kinase C) and MAPK (mitogen-activated protein kinases) and in the nucleus such as expression of c-fos, c-jun and other genes. Microgravity severely affects also the cytoskeleton. However, the primary molecular mechanisms how microgravity influences cell signaling are unknown.

Microgravity also impaired monocyte function: During the spacelab-mission SLS-1 monocytes lost their capability of secreting IL-1 [25] and of expressing IL-2-receptor [26]. However, the molecular mechanisms are not identified. Examination of gene expression of monocytes under real microgravity demonstrated significant changes in gene induction associated with differentiation of monocytes into macrophages [27]. Kaur *et al*. [28] investigated monocytes isolated from astronauts before and after a mission and compared the results with control groups. They found a reduction of phagocytosis and a reduced *oxidative burst- *and degranulation-capacity. Meloni *et al*. [29] recently demonstrated that simulated weightlessness leads to massive alterations in the cytoskeleton of monocytes, which in turn influences motility and recently revealed during an ISS experiment a severe reduction in the locomotion ability of monocytic cells in microgravity [30]. Importantly, LFA-1 and ICAM-1 adhesion proteins expression seemed also to be sensitive to microgravity, whereas their interaction is not altered [30]. It seems that not all cell types of the immune system are sensitive to reduced gravity: Extensive studies with natural killer cells in simulated weightlessness and in real microgravity on board of the ISS revealed that neither cytotoxic effects nor interferon production is altered in microgravity [31]. Major gravi-sensitive signal transduction elements in mammalian cells are summarized in additional file 1 and figure 1.

## Cell migration in microgravity

*Neutrophil granulocytes *demonstrate the body's first line of host defense by recognizing and killing microorganisms. Neutrophil locomotion is integral for immune effector function, because the cells have to leave the blood vessels and navigate to places of infection and injury to fulfill their main task of phagocytosis. They are one of the most important cells regulating the immune response, because they can influence both induction and effector stage of immune reactions. Several studies provided evidence of a disturbed function of neutrophil granulocytes: Returning astronauts of spaceflight missions exhibited a strong increase of neutrophil granulocytes immediatedly after landing [32,33], and neutrophil chemotactic assays showed a 10-fold decrease in the optimal dose-response after landing [34]. In a parabolic flight experiment neutrophil granulocytes showed a dramatic increase of the superoxide-anion production [35]. Whereas some studies discuss an influence of space flights on the neutrophil phagocytotic activity and oxidative function [32], the influence of gravity on the migration of neutrophil granulocytes, which also determines the efficiency of an immunologial response, is still not known.

*Cell migration *is an essential characteristic of life. Multicellular organisms must be motile to obtain nourishment, evade being eaten in their own right, respond to environmental changes and reproduce. Likewise, unicellular organisms such as Paramecium or Loxodes must dynamically respond to fluctuations in ever-changing surroundings to assure survival [6]. However, cell migration is also an essential characteristic of many normal and abnormal biological processes within the human organism including embryonic development, defense against infections, wound healing and tumor metastasis [36,37]. In previous studies using simulated microgravity, changes in gravity demonstrated an inhibition of lymphocyte locomotion through type I collagen [38,39], and culture of human bone marrow CD34+ cells using NASA 's rotating wall vessels resulted in a decreased migration potential [40]. An altered movement in real microgravity was shown for leukocytes and Jurkat T cells, too [41,42], whereas the underlying signal transduction mechanisms are still illusive. On the other side, T cells become more motile after being cultured in 10 g hypergravity [43].

The *cytoskeleton *is responsible for giving a cell its shape and for generating the forces required for cell motility. It is an internal network of at least three types of cytosolic fibers: actin filaments, microtubules and intermediate filaments. Actin, one of the most highly conserved and abundant eukaryotic proteins, is constantly polymerized and depolymerized within cells to invoke cellular motility, tissue formation and repair [44,45]. Actin dynamics are considered to be the major component of the cytoskeleton responsible for cell motility. It has been shown to be essential for the migration of T lymphocytes as well as neutrophil granulocyte migration, a conclusion readily assumed as actin-depolymerizing drugs inhibit cellular motility [46,47]. In contrast, an intact microtubule network does not appear to be required for neutrophil migration, because microtubule-disrupting drugs such as colchicine even induce the migration of neutrophils [48], probably by inducing changes in the actin network.

## Gravisensitivity of the cytoskeleton

Multiple investigators have reported that this complex network of fibers is sensitive to environmental factors such as microgravity and altered gravitational forces [49]. Several studies demonstrate modifications of the actin and microtubule cytoskeleton in microgravity. Already a few minutes of simulated weightlessness provided by 2D-clinorotation affected the cytoskeleton of lymphocytes, astrocytes, neurons and glial cells, disorganizing microtubules, intermediate filaments and microfilaments [50,51]. Morphological differences of both the microtubule and actin components of the cytoskeleton have been observed in cells grown in real and simulated microgravity [50,52]. Gruener and Hughes-Fulford reported that actin reorganization responded to the gravity level and showed abnormal assembly of actin stress fibers during spaceflight [53-55]. In human mesenchymal stem cells F-actin stress fibers were disrupted within three hours of initiation of modeled microgravity [56]. On the contrary, in Jurkat cells microgravity did not change the structure of actin but from vimentin [42]. Other studies have shown that microtubules are gravity sensitive, too [57]. Microtubule self-assembly is inhibited in the absence of gravity in space [58], and Lewis et al. observed that the microtubule filaments extended from a poorly defined centrosome in human Jurkat cells [52]. Moreover, cancer cells grown under microgravity exhibited an increased and highly disorganized vimentin as well as altered microtubules [59,60].

Many components of *signal transduction pathways *are known to regulate the cytoskeleton [11,52,54]. With regard to migration, neutrophils are the fastest moving cells at all with a speed maximum of 15 to 20 μm/min [61], and the starting signal for their migration to sites of inflammation is provided by early proinflammatory cytokines such as the bacterial peptide N-Formyl-L-methionyl-L-leucyl-L-phenylalanine (fMLP) [62]. The bacterial peptide fMLP is the major chemotactic peptide produced by *Escherichia coli *and known to be a strong stimulator for the migration of neutrophil granulocytes. fMLP binds and activates a class of G-protein-coupled receptors. Ligand binding leads to the activation of two signalling pathways: (i) the activation of the PLC-gamma generates inositol-1,4,5-phosphate (IP_3_) and diacylglycerol (DAG), which results in IP_3 _mediated release of intracellularly stored calcium in the endoplasmatic reticulum and DAG-mediated activation of the protein kinase C (PKC). These are key events for the regulation of locomotory activity [62-64].(ii) the activation of the adenylyl cyclase leads to an increase of cytosolic cAMP, which results in an activation of the sarcoplasmatic/endoplasmatic reticulum calcium ATPase (SERCA) pump and calcium sequestration. Thus, stimulation of neutrophils with fMLP activates a signal transduction pathway ultimately leading to an elevation of cytosolic calcium which has been shown to be essential for the development of actin-based migration [65]. In addition, observations of migrating neutrophils within a three-dimensional collagen matrix revealed a frequent increase of calcium in those parts of the cells that underwent shape changes a few seconds later, and visualization of the calcium signal was shown to be a directionality marker for the orientation of neutrophils locomoting in a three-dimensional space [62]. With regard to cell migration, the inhibition of lymphocyte locomotion observed under microgravity culture conditions could be reversed by prior activation with phorbol myristate acetate (PMA), which directly activates the PKC [39].

Migration of immune cells is a crucial process during a multitude of physiological and pathophysiological conditions such as development, defense against infections and wound healing [36,37]. Leukocytes move through the body in order to keep the organism under immunological surveillance and to respond to pathogenic invading microorganisms. Migration within the body tissues and through endothelial barriers is strongly dependent and regulated both by cytoskeletal processes and by expression of surface adhesion molecules such as selectins and integrins [66], which interact with components of the extracellular matrices. Whereas the influence of microgravity on the cytoskeleton is well investigated [49], there is only little known about adhesion molecule expression in altered gravity. Importantly, the phenomenon of altered cytoskeletal organisation and migration in microgravity has been described well in non-adherent cells so far, but there is only little knowledge of cytoskeletal organisation in adherent cells, such as endothelial cells. Experiments on board of the Space Shuttle Mission STS-57 revealed a decrease of selectin-expression, but no change in ICAM-1 expression in splenocytes [10]. Moreover, long-term gravity vector changes modulate expression of ICAM-1, E-selectin and VCAM-1 on cultured endothelial cells, and increased adhesion of PMA-activated lymphocytes on endothelial monolayers in simulated and in real microgravity [67]. An experiment, which addressed the focal adhesion in connective tissue in microgravity, has been performed on board of STS-107 Spacelab [68], but got lost due to the fatal accident of the Space Shuttle Columbia in 2003. Thus, clear results about adhesion molecule expression after onset of altered gravity are still missing.

It is possible that the molecular and cellular structure of life on Earth may require gravity for survival, either in individual or in evolutionary terms, and it is therefore possible that exactly such gravity-dependent or gravity-sensing mechanisms will keep us dependent from the gravity field of Earth. No one can really neglect the importance of gravity on biological systems and only the facts that research platforms are rare and that access to altered gravity is limited, reduce the speed of progress in gravity research compared to other disciplines.

Technically, we are able to travel to Earth orbit or Moon for weeks up to months, and most probably, in the next decades we will be able to fly to Mars. But until now there is only limited knowledge about the biological and biomedical effects of weightlessness on organisms and humans, especially on the cellular and molecular level, where therapeutic or preventive countermeasures could be developed.

## Competing interests

The authors declare that they have no competing interests.

## Supplementary Material

Additional file 1 **Gravi-sensitive signal transduction elements in mammalian cells.** The figure summarizes known gravi-sensitive signal transduction elements in mammalian cells. Please note, that the primary molecular mechanisms how microgravity influences cell signaling, are still unknown. Click here for file
