# Supplementary material for: Signal transduction in cells of the immune system in microgravity
Source: Cell Commun Signal. 2008 Oct 28;6:9. doi: 10.1186/1478-811X-6-9 (PMC2583999; doi:10.1186/1478-811X-6-9)
Supplement: Additional file 1 — Gravi-sensitive signal transduction elements in mammalian cells. The figure summarizes known gravi-sensitive signal transduction elements in mammalian cells. Please note, that the primary molecular mechanisms how microgravity influences cell signaling, are still unknown. [file 1478-811X-6-9-S1.doc]

**Table 1: Gravi-sensitive signal transduction elements in mammalian cells**

protein kinase C lymphocytes [11, 12]

NF-kB and MAPK-signaling lymphocytes [14]

*c-fos, c-myc, c-jun* osteoblasts [15]

fas, p53, bax, bcl-2 human thyroid carcinoma cells, osteoblasts [17, 19, 21]

IL-2 receptor T lymphocytes [22, 23,26]

IL-1 monocytes [25]

Cytoskeleton monocytes, lymphocytes, astroglia cells, myocytes, [29,42,49,50-57,59,60]

mesenchymal stem cells, thyroid carcinoma cells, breast cancer cells

selectin-expression lymphocytes [10]

ICAM-1, E-selectin, endothelial cells [67]

VCAM-1 expression
